# Purification and Characterization of Antibacterial Activity against Phytopathogenic Bacteria in Culture Fluids from *Ganoderma lucidum*

**DOI:** 10.3390/molecules26185553

**Published:** 2021-09-13

**Authors:** Loreto Robles-Hernández, Nora A. Salas-Salazar, Ana C. Gonzalez-Franco

**Affiliations:** Facultad de Ciencias Agrotecnológicas, Universidad Autónoma de Chihuahua, Ciudad Universitaria S/N Campus 1, Chihuahua 31310, Mexico; lrobles@uach.mx (L.R.-H.); nsalas@uach.mx (N.A.S.-S.)

**Keywords:** antibacterial polysaccharides, GC-MS, basidiomycetes, HPLC-APCI-MS

## Abstract

Previous studies of *Ganoderma lucidum* have focused on its medicinal applications. Limited information is available about its antibacterial activity against plant pathogens. Thus, the goal of this study was to purify and characterize the antibacterial activity against plant pathogenic bacteria from culture fluids of *G. lucidum.* The nature of the bioactive components was determined using heat boiling, organic solvents, dialysis tubing, gel exclusion chromatography (GEC), proteinase sensitivity, HPLC, HPLC-APCI-MS, and GC-MS. The bioactive compounds were neither lipid, based on their solubility, nor proteic in nature, based on proteinase digestion and heat stability. The putative-bioactive polysaccharides have molecular weights that range from 3500 to 4500 Daltons as determined by dialysis tubing, GEC and APCI-MS analysis. The composition of the antibacterial compounds was determined by GC-MS. This is the first report of small polysaccharides produced by *G. lucidum* with activity against bacterial plant pathogens.

## 1. Introduction

*Ganoderma lucidum* is a basidiomycete mushroom used in oriental medicine for its immunomodulating activity [1,2]. This immunomodulating action is mediated by polysaccharides, especially β-d-glucan [2,3,4,5,6,7,8], triterpenoids [9], and proteins, including LZ-8 [10]. The major effects of these active substances include mitogenicity and activation of immune effector cells, such as T cells, macrophages, and natural killer cells. This activity, in turn, spurs the production of cytokines, including interleukins, tumor necrosis factor-α, and interferons [11].

In addition to the immunomodulation, some *Ganoderma* species are bactericidal. Crude extracts of *Ganoderma* species (*G. lucidum*, *G. pfeifferi*, and *G. resinaceum*) inhibit growth of *Bacillus subtilis* [12]. Two secondary metabolites, the triterpenes ganomycin A and ganomycin B, from *G. pfeifferi* inhibit Gram-positive strains, such as *B. subtilis*, *S. aureus*, and *Micrococcus flavus* [13]. However, research on ganomycins has primarily focused on their anti-tumor ability.

*Ganoderma applanatum* produces the sterols 5α-ergost-7-en-3β-ol, 5α-ergost-7, 22-dien-3β-ol, 5, 8-epidioxy-5α-ergost-6, 22-dien-3β, and a novel lanostanoid that are active against Gram-positive bacteria, including *Bacillus cereus* and *S. aureus*. These sterols also inhibited Gram-negative bacteria, such as *E. coli*, and *Pseudomonas aeruginosa* [14,15]. *Ganoderma oregonense* produces a crystalline substance active against Gram-positive bacteria [16]. Previous studies of *G. lucidum* have mainly concentrated on its pharmaceutical properties. Limited information is available on the ability of this fungus to inhibit bacterial plant pathogens. Bioactive extracts of *G. lucidum* were tested against isolates of *Xantomonas campestris* pv. *vesicatoria* that cause bacterial spot disease in pepper; culture fluids inhibited 100% of the phytopathogenic isolates in in vitro assays and reduced foliar infection and bacterial populations in greenhouse experiments [17]. Thus, the goal of this study was to purify and characterize the active antibacterial fraction present in culture fluids of *G. lucidum* against plant pathogenic bacteria. We found that the antibacterial activity of the bioactive culture fluids of *G. lucidum* was not affected by long term storage, heat boiling, or proteinase digestion treatments. The novel antibacterial compounds were polysaccharides of low molecular weight and have d-glucose, D- ribose, and β-d-galactofuranose as main components.

## 2. Results

### 2.1. Determination of Bioactive Culture Fluid Stability

Culture fluids of *G. lucidum* were tested in three forms: freshly harvested, boiled for 60 min, and stored for 90 days at room temperature. The freshly harvested culture fluids inhibited the growth of almost all the bacteria tested (Table 1). No activity was observed against *Burkholderia cepacia* or *Pseudomonas fluorescens*. Slight inhibition was observed with *Brenneria quercina* and *Rathayibacter tritici*. Culture fluids that were stored for 90 days at room temperature or boiled for an hour had similar antibacterial activity, but, after boiling, the slight growth inhibition was not observed with *R. tritici* or *Brenneria quercina* (Table 1).

### 2.2. Solubility Determination of Bioactive Compounds

**Figure 1:**
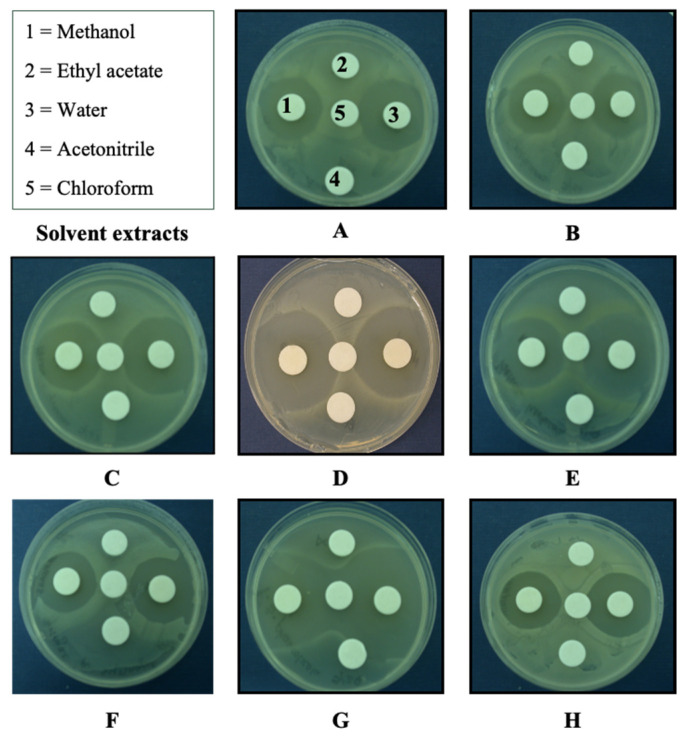
Antibacterial activity of extracted freeze-dried culture fluids of *Ganoderma lucidum* using five different solvents. Test bacteria: **A** = *Acidovorax avenae*, **B** *= Agrobacterium tumefaciens*, **C** = *Brenneria quercina*, **D** = *Erwinia carotovora* subsp. *carotovora*, **E** = *Pantoea herbicola*, **F** = *Pseudomonas syringae* pv. *syringae*, **G** = *Pseudomonas syringae* pv. *phaseolicola*, and **H** = *Xanthomonas campestris* pv. *campestris*.

Solubility was the first step towards isolating the antibacterial components from *Ganoderma lucidium* culture fluids. After extracting freeze-dried culture fluids with chloroform, ethyl acetate, acetonitrile, methanol, and water, the extracts were loaded separately onto disks and evaporated under aseptic conditions for antibacterial bioassays. Only the methanol and water extracts showed inhibition of all the bacteria tested. However, *Erwinia carotovora* subsp. *carotovora* and *P. syringae* pv. *phaseolicola* were the most inhibited, whereas *P. syringae* pv. *syringae* was least inhibited (Figure 1).

### 2.3. Proteinase K Does Not Affect Antibacterial Activity

Proteinase K digestions eliminated proteic compounds from *G. lucidum* culture fluids but did not eliminate antibacterial activity (Table 2). For example, *E. carotovora* subsp. *carotovora* did not grow on nutrient agar plates containing sterilized culture fluids from potato dextrose broth culture (1:1) nor plates amended with proteinase K-treated culture fluids. Proteinase K-treated culture fluids did show increased antibacterial activity against *X. campestris* pv. *campestris*.

### 2.4. Size Determination of the Bioactive Compounds

**Figure 2:**
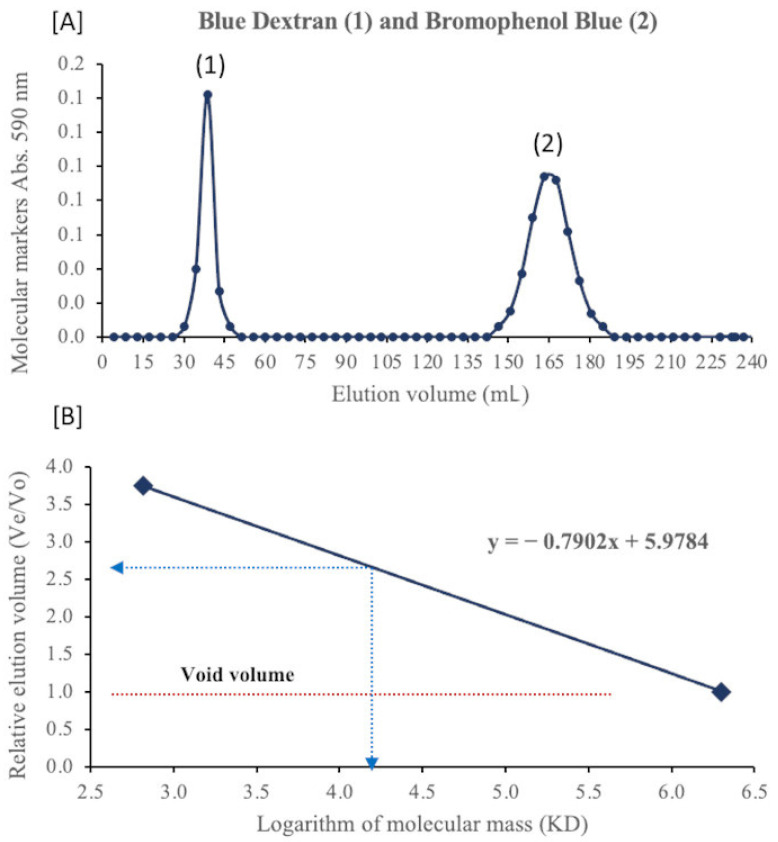
Elution diagram of a mixture of [**A**] molecular markers on Sephadex G-75 in a 0.05 M phosphate buffer, pH 7.2 and [**B**] relative elution volume of antibacterial fraction versus molecular weight for Bromophenol Blue and Blue Dextran.

Using dialysis tubing, antibacterial activity was detected in the water wash, but not in the tubing content. Thus, the antibacterial compounds were approximated to be smaller than 3500 Daltons (Table 2). To confirm the size estimated by dialysis, gel exclusion chromatography was used. A Sephadex G-75 column was calibrated (Figure 2A) with a mixture of molecular markers containing 0.03% Bromophenol Blue (BPB, MW = 669) and 2% of Blue Dextran (BD, MW = 2,000,000). Void volume (Vo), elution volume (Ve), and relative elution volume (Ve/Vo) were determined for BD and Ve and Ve/Vo for BPB. The Vo, Ve, and Ve/Vo for BD were 38 mL, 38 mL, and 1.0, respectively; and, for BPB, the Ve and Ve/Vo were 165 mL and 3.75 mL, respectively. The bioactive fraction of culture fluids from gel exclusion chromatography yielded a Ve and Ve/Vo of 122 mL and 2.54 mL, respectively. Plotted as a standard curve, the molecular mass of the bioactive fraction was between 4000 and 4500 Daltons (Figure 2B).

### 2.5. HPLC Analysis

**Figure 3:**
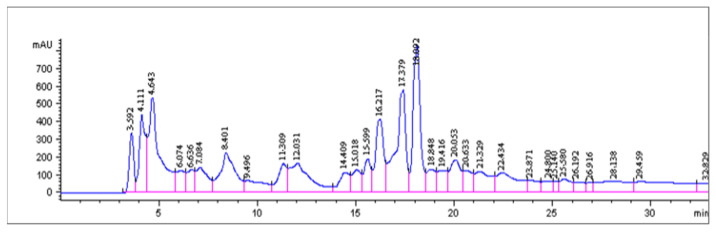
HPLC chromatogram of bioactive fraction after gel exclusion chromatography.

Using HPLC, the bioactive methanol extracts of culture fluids showed at least 12 peaks of interest, but the first three peaks overlapped (Figure 3). When eight fractions representing these peaks were collected, freeze dried, and tested for biological activity, only fraction one, the first three overlapped peaks (left to right), showed antibacterial activity.

#### HPLC-APCI-MS Analysis

**Figure 4:**
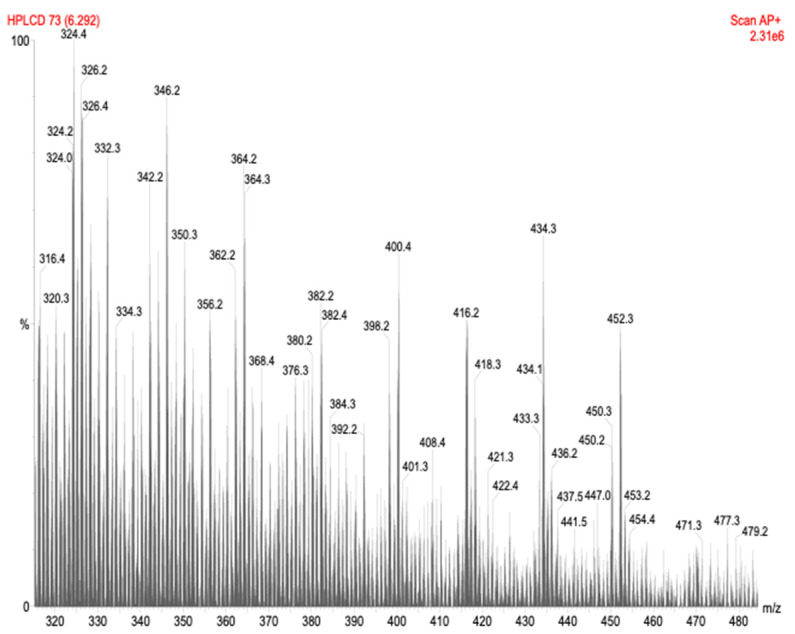
APCI-MS spectra of the bioactive fraction after HPLC separation. A consistent peak separation of 18 Daltons is observed.

The bioactive fraction (fraction one) further analyzed using high performance liquid chromatography-atmospheric pressure chemical ionization-mass spectrometry (HPLC-APCI-MS) yielded a consistent peak separation of 18 Daltons apart (Figure 4). Such fractionation is common among polypeptides and polysaccharides, where successive water molecules are released. Since the bioactive compounds are neither lipids nor proteic compounds as demonstrated with solvent extraction, Proteinase K digestions, heat boiling, and the APCI-MS results strongly support that the bioactive fraction contains polysaccharides with an approximate molecular weight from 3000 to 4500 Daltons.

### 2.6. GC-MS Analysis

**Figure 5:**
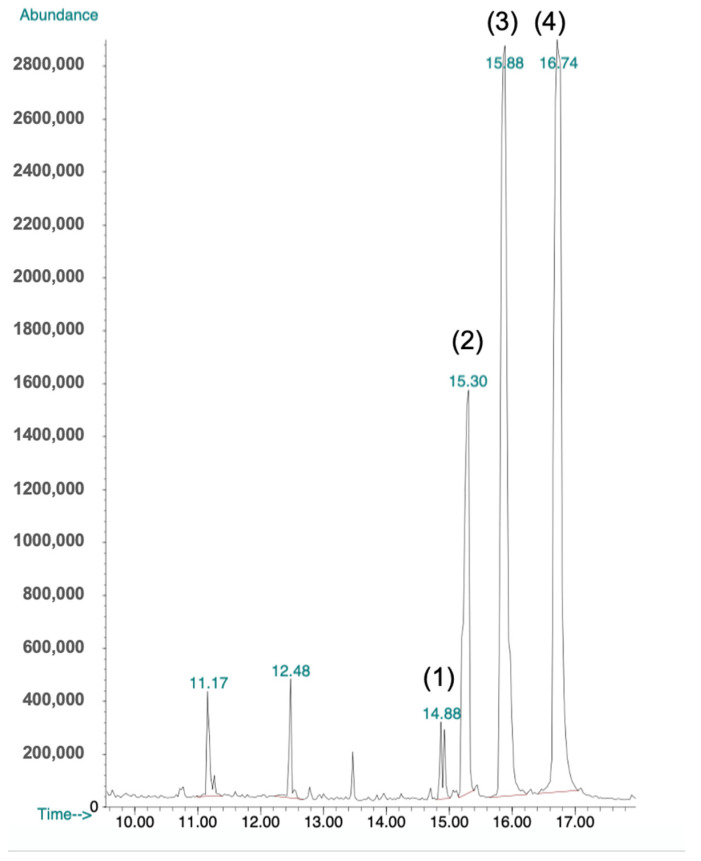
GC-MS chromatogram of bioactive fraction separated in a reverse phase HPLC. Peaks 1, 2, 3, and 4 were evaluated for chemical composition.

**Figure 6:**
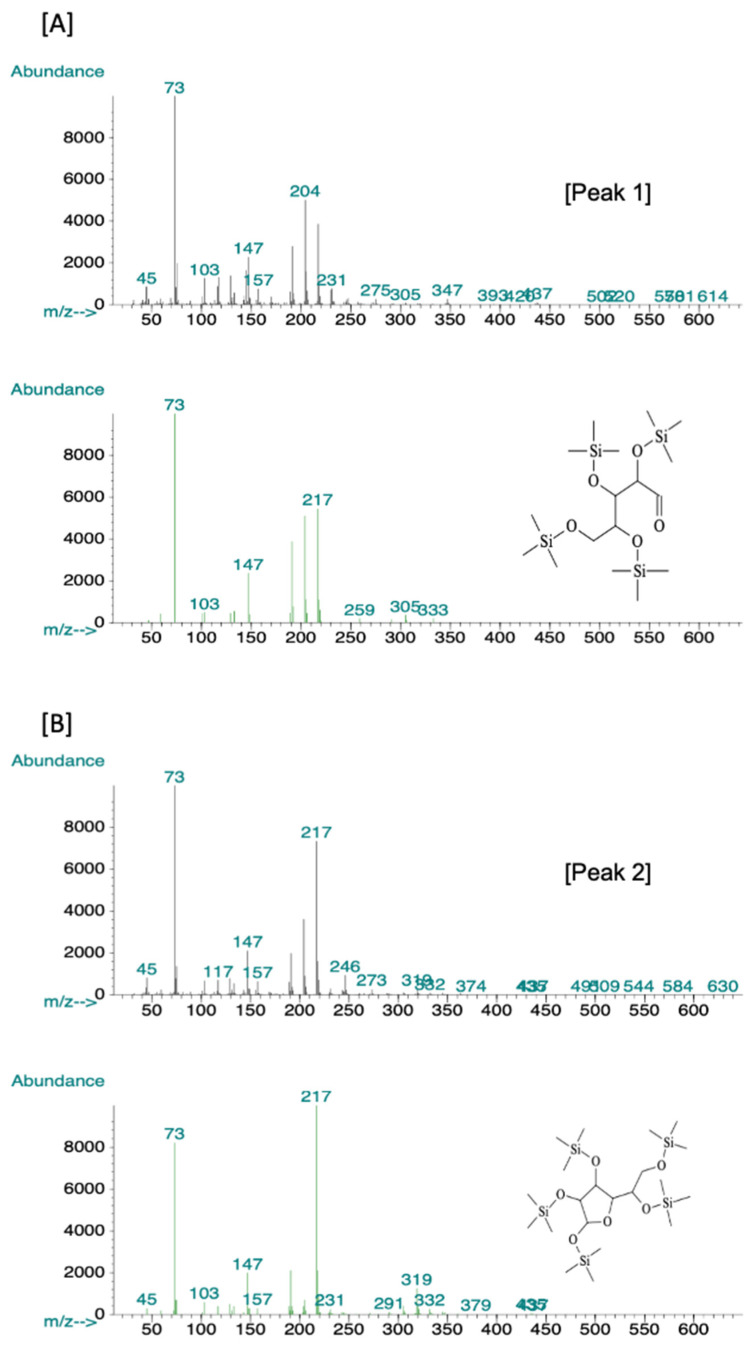
GC-MS spectrum of peak 1 and 2 selected from GC-MS chromatogram and compared with the library of mass spectra stored in the Chemstation database of MS. Comparison showed that peak 1 is 91% similar to D-ribose [**A**], and peak 2 is 80% similar to β-d-galactofuranose [**B**].

**Figure 7:**
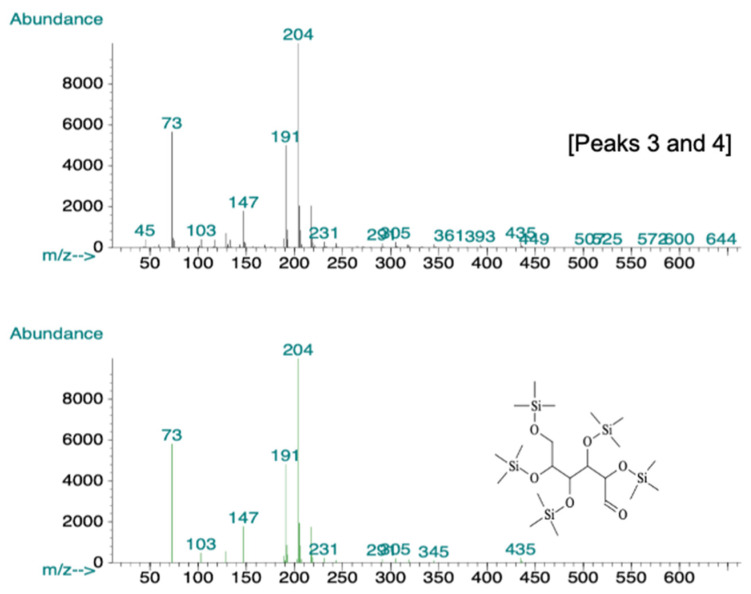
GC-MS spectrum of peak 3 and 4 selected from GC-MS chromatogram and compared with the library of mass spectra stored in the Chemstation database of MS. Comparison showed that both peaks were 95% similar to d-glucose.

To confirm and characterize the presence of polysaccharides in the bioactive fraction, gas chromatography-mass spectroscopy (GC-MS) analysis yielded four peaks (Figure 5). The peaks at 14.88, 15.30, 15.88, and 16.74 were compared with known spectra in the Wiley and National Institute of Standards and Technology library of mass spectra in the Chemstation database (approximately 200,000 spectra). The peak at 14.88 had 91% similarity to d-ribose (Figure 6A), the peak at 15.30 had 80% similarity to β-d-galactofuranose (Figure 6B), and the last two peaks were 95% similar to d-glucose (Figure 7).

## 3. Discussion

Although previous studies of *G. lucidum* mainly focused on its medicinal properties, we found that culture fluids of *G. lucidum*, inhibited plant pathogenic bacteria, including gram-positive and gram-negative bacteria. Since this antibacterial activity was stable under long term storage and to heat, it is unlike most other antibiotics or peptide-based toxins. In addition, the bioactive compounds are not proteic in nature based on Proteinase K digestions or lipid based on their solubility; the antibacterial components are most likely polysaccharides.

Furthermore, our data suggests that *G. lucidum* produces novel antibacterial polysaccharides based on their separation by APCI-mass spectroscopy (Figure 4). This kind of fractionation is common in the ionization of polysaccharides, where successive molecules of H_2_O (MW = 18) are released as large molecules of polysaccharides are fragmented [18,19]. The APCI spectrum suggests their molecular weight to be from 3000 to 4500 Daltons consistent with the results from dialysis and gel exclusion chromatography (GEC).

Polysaccharides, in bioactive fraction one, were confirmed with GC-MS analysis, which showed the main components to be d-glucose, D- ribose, and β-d-galactofuranose (Figure 6 and Figure 7). The compounds are water-soluble, and no bioactive proteic compounds were involved in the observed antibacterial activity.

Numerous bioactive glycosides of plant, fungi, and bacteria have been reported [20,21]. The most studied antimicrobial glycosides are tuliposides A and B, isolated from tulips, which have activity against *B. subtilis* [20]. Clavicepcin from *Claviceps purpurea* has antimicrobial activity, and its hydrolysis yields molecules of glucose and manitol [21]. Streptomycin produced by some *Streptomyces* species is an aminocyclitol glycoside antibiotic, which disrupts ribosomal RNA and interferes with prokaryotic protein synthesis [22].

Numerous bioactive polysaccharides have been isolated from fruiting bodies and culture fluids of *G. lucidum* [9,15]. The most common polysaccharides have been isolated from fruiting bodies Fl-1a (β-glucan), FIII-2b (hetero-β-glucan), acid heteroglucan, and chitin xyloglucans; others, such as β-glucans, have been isolated from culture fluids [9]. Most of these polysaccharides have molecular weights that vary from 4 × 10^5^ to 1 × 10^6^, and they have been classified as β-d-glucans [23]. β-d-glucan structure has been elucidated using NMR, X-ray crystallography, methylation, periodic acid oxidation, and enzymatic hydrolysis. Using X-ray diffraction, β-d-glucans display a secondary right triple strand helix [24] that consists of a linear backbone of β (1→3)-linked d-glucopyranosyl groups branched from the C6 position. Branches are usually composed of one or more glucose residues. The biological activity of β-d-glucans is influenced by their solubility in water and their molecular size [22].

Our novel polysaccharides differ in size and biological activity from those previously reported from *G. lucidum*. These novel polysaccharides are much smaller than other reported polysaccharides and have antibacterial activity against gram-negative and gram-positive plant pathogenic bacteria. The other bioactive polysaccharides in *G. lucidum* have medicinal applications, such as immunomodulators, anti-tumor, and bactericidal agents [1,12,13,15]. Like other bioactive polysaccharides in *G. lucidum*, our novel polysaccharides are water-soluble and are mainly composed of d-glucose.

Our work is one of the first reports on the antibacterial activity of *Ganoderma lucidum* against plant pathogens, including gram-positive and gram-negative bacteria. These novel antibacterial compounds are heat stable, polysaccharide in nature with their main components being d-glucose, D- ribose, and β-d-galactofuranose, and with approximate molecular weights ranging from 3000 to 4500 Daltons. Further work is required to elucidate the mode of action of these novel antibacterial polysaccharides.

## 4. Materials and Methods

### 4.1. Microbial Strains and Culture Conditions

*Ganoderma lucidum* was maintained on rich solid medium (RSM) containing 3 g of yeast extract, 200 g of potato infusion, 30 g of malt agar, 1 g of Bacto peptone, 60 g of sucrose, 15 g of agar, 1 tsp of molasses, and 3.75 g of ground oat meal per liter. All plant pathogenic bacteria, including *Agrobacterium tumefaciens*, *Agrobacterium* rhizogenes, *Acidovorax avenae*, *Burkholderia cepacia*, *Erwinia carotovora* subsp. *carotovora*, *Pseudomonas fluorescens*, *Pantoea herbicola*, *Rathayibacter tritici*, *Pseudomonas syringae* pv. *phaseolicola*, *Pseudomonas syringae* pv. *syringae*, *Pseudomonas corrugate* 0782-6, *Xanthomonas campestris* pv. *translucens*, and *Xanthomonas campestris* pv. *campestris* were maintained in nutrient agar (NA, Difco Laboratories, Detroit, MI, USA). All bacterial pathogens and the *Ganoderma lucidum* strain were kindly provided by Dr. W.C. Chun from the Laboratory of Bacteriology, Department of Entomology, Plant Pathology and Nematology at the University of Idaho, Moscow, Idaho, USA. For long-term storage, bacterial cultures were stored in Luria Broth with 20% glycerol at −80 °C. *G. lucidum* was preserved under the same conditions, but in Potato Dextrose Broth medium (Difco Laboratories, Detroit, MI, USA).

### 4.2. Production of Bioactive Culture Fluids

*Ganoderma lucidum* cultures were grown in stationary 2000 mL Erlenmeyer flasks containing 200 mL of potato dextrose broth. Flasks were inoculated with 5 pieces (cut with a 1 cm diameter No. 8 core borer) of actively growing mycelia from 14 day-old RSM cultures and incubated at 30 °C for 21 days. Mycelial growth was removed by centrifugation (14,000 rpm, 15 min, 5 °C). Culture fluids were collected and sterilized under vacuum with 0.22 μm membrane filters (Membrane Filters, Isopore^TM^, Wicklow, Ireland), freeze-dried, and stored at room temperature in a desiccator until use.

### 4.3. Antibacterial Activity

Two methods were used to determine antibacterial activity:

(1) The agar dilution method (poisoned agar) [25,26] was used to evaluate the antibacterial activity in two assays: culture fluid stability and Proteinase K sensitivity. The method consisted of incorporating in a 1:1 ratio the treatment (treated culture fluids) into a molten twofold concentrated NA medium and poured into 90 × 15 mm petri plates. The inoculation (15 μL) of the bacterial test at a concentration of 10^7^ colony forming units (CFU)/mL was spotted onto the agar plate surface. Plates were incubated at 28 °C for 24 h. A mix of potato dextrose broth and twofold concentrated NA medium in a 1:1 ratio was poured into petri dishes to confirm the growth of the bacteria (control treatment). Plates were incubated at 28 °C for 24 h. Results were recorded as (+) = growth inhibition, (±) = partial growth inhibition, and (−) = no growth inhibition. Each experiment was repeated three times.
(2) The disk diffusion method [25,27] was used to evaluate antibacterial activity using the following assays: solubility of the bioactive compounds (testing different solvents), dialisis tubing, and bioactive fractions in the gel exclusion chromatography and HPLC. The method consisted of loading onto the paper disks (9 mm diameter) separately four times with 100 μL of the culture fluid samples and drying them between each application under aseptic conditions. Loaded disks were then evenly distributed on the bacterial lawn of NA plates. Bacterial lawns were made by spreading 100 μL of bacterial suspensions at 10^7^ colony forming units (CFU)/mL. Plates were sealed with parafilm and incubated at 28 °C. After 24 h, inhibition zone diameters were recorded. Each experiment was repeated three times.

The preparation of bacterial solution in both methods was the same. Phytopathogenic bacterial growth of one to two-day-old nutrient agar plates were collected with a sterile loop and suspended in sterile saline solution (0.85% NaCl, pH = 7.0 ± 0.1). Serial dilution protocols were performed to adjust the concentration at 10^7^ CFU/mL.

### 4.4. Determination of Bioactive Culture Fluid Stability

To check stability for antibacterial activity of filter-sterilized potato dextrose broth culture fluids, three forms were tested: freshly harvested, boiled for 60 min, and stored for 90 days at room temperature. Nutrient Agar and potato dextrose broth in a 1:1 ratio was used as control. Antibacterial activity was determined using the poisoned agar as described in Section 4.3. To evaluate the solvent effect on bacterial growth, only the solvents were loaded and dried on the disks as controls and distributed evenly onto NA plates previously seeded with test bacteria; no growth inhibition was detected.

### 4.5. Solubility of Antibacterial Compounds

The solubility of bioactive compounds was conducted in chloroform, ethyl acetate, acetonitrile, methanol, and water. Organic solvent extractions involved mixing 1 g of freeze-dried culture fluids in 2 mL of solvent and shaking at 225 rpm for 2 h. After centrifuging at 18,626× *g* for 8 min, the supernatant was collected and stored at −20 °C until use. Antibacterial activity was detected on lawns of test bacteria as described in Section 4.3. To evaluate the solvent effect on bacterial growth, only the solvents were loaded and dried on the disks as controls and distributed evenly onto agar plates previously seeded with test bacteria; no growth inhibition was detected.

### 4.6. Proteinase-K Sensitivity

Proteinase K (Sigma Chemical Company, St. Louis, MO, USA) was used to determine if the antibacterial activity was proteinaceous. Enzyme solutions were prepared according to the manufacturer. Three milliliters of culture fluids were treated with 300 μL of Proteinase K (1 µg/mL dissolved in 30 mM Tris-HCl, 10 mM EDTA, 1% SDS (pH 8.0)) and incubated for 2 h in a 40 °C water bath. Treatments were prepared and analyzed for antibacterial activity using the method of poisoned agar as described in Section 4.3. Proteinase K digestion was confirmed by sodium dodecyl sulfate polyacrylamide-gel electrophoresis (SDS-PAGE) using the method of Laemmli [28] (data not shown).

### 4.7. Size Determination

#### 4.7.1. Dialysis Tubing

Membrane tubing with a molecular weight cut-off of 3500 (Spectrum Medical Industries, Inc., Los Angeles, CA, USA) was prepared according to the manufacturer. Ten mL of culture fluids were dialyzed against double distilled water (1000 mL) overnight with stirring in a beaker at 4 °C. The volume of both tubing and beaker content were adjusted to a final volume of 10 mL with a rotary evaporator or with addition of sterile double distilled water [29]. Both tubing and beaker contents were tested for antibacterial activity on lawns of test bacteria as described in Section 4.3.

#### 4.7.2. Gel Exclusion Chromatography

In addition to dialysis tubing, gel exclusion chromatography was performed using Sephadex G-75 (Sigma Chemical Company, St. Louis, MO, USA). Ten grams of resin were dissolved in a 0.05 M phosphate buffer pH 7.2 with occasional stirring. The resin, allowed to hydrate overnight at 4 °C, was packed in a glass column (90 cm × 1.5 cm). The column was equilibrated three times with the phosphate buffer and the operating flow rate adjusted. The column was calibrated using a mixture of molecular markers, including 0.03% Bromophenol Blue (MW = 669) and 2% Blue Dextran (MW = 2,000,000). The void volume (Vo), elution volume (Ve), and the relative elution volume (Ve/Vo) were determined [30,31]. Since the large molecules of Blue Dextran do not pass through Sephadex (G-75), its Ve and Vo are equal (Figure 2A). Culture fluids (10 times concentrated) were loaded as 2.5% of the total bed volume. The operating flow rate was 0.33 mL/min, volume collected per fraction 4.3 mL, running time 14 h, and running temperature 4 °C. Fractions were collected, freeze-dried, and tested for antibacterial activity as described in Section 4.3 by the disk diffusion method with a small variation; the bioassays were performed in polystyrene plastic plates (24-well, diameter 16 mm) (Corning, St. Louis, MO, USA). After determination of the bioactive fraction, its Ve and Ve/Vo were determined.

### 4.8. Reverse Phase HPLC Separation of Methanol Soluble Bioactive Fraction

A Hewlett-Packard Series II 1090 liquid chromatograph with a diode array detector (Hewlett-Packard, Avondale, PA, USA) equipped with a Phenomenex Luna C-8 100 A (150 × 4.6 mm 5 μ) column was used. The HPLC was operated in a gradient mode from 0–8 min, 100% water. An isocratic gradient was applied from 8–25 min to reach 100% methanol. From 25–28 min, 100% methanol. From 28–33 min, 100% water with an operating flow rate of 0.5 mL/min. The injection volume was 8 μL. The bioactive methanol extracts were dried with a rotary evaporator and dissolved in sterilized double distilled water for HPLC analysis. Fractions were collected, freeze dried, and tested for antibacterial activity as described in Section 4.3.

#### HPLC-APCI-MS Analysis of the Bioactive Fraction

The bioactive fractions were analyzed for molecular weight and composition using high performance liquid chromatography-atmospheric pressure chemical ionization-mass spectrometry (HPLC-APCI-MS) (Quattro II, Micromass). Samples were injected directly in a reverse phase HPLC (HP-1090) with a flow rate of 0.6 mL/min. A potential of 3.26 kV was applied to the APCI corona. The sample cone voltage was maintained at 25 V. The counter electrode, skimmer, and RF lens potentials were tuned to maximize the ion beam for the given solvent. Detection resolution was set at 15,000, and source block temperature was constant at 150 °C and APCI probe temperature at 300 °C. The instrument was calibrated using polyethylene glycol solution.

### 4.9. GC-MS Analysis of the Antibacterial Fraction

Bioactive fractions were separated in a reverse phase HPLC in a 10 mM NaH_2_PO_4_ solvent. Peaks were collected, freeze dried, and derivatized with trimethylchlorosilane. Derivatizations were performed in glass HPLC vials and analyzed in a HP 5890 Series II gas chromatograph equipped with an HP 6890 Series auto-injector and a Phenomenex ZB5 capillarity column (30 m × 0.25 mm × 0.2 μm), coupled with an HP 5989A mass spectrometer. Injector temperature was 250 °C, and the initial and final oven temperature was 100 °C. The HP quadrupole MS was controlled by HP-MS Chemstation software. The analysis of 3 μL samples was under the following conditions: repeller, 7 V; emmision, 300 V; electron energy, 70 eV. The source temperature was 226 °C, and the quadrupole temperature was 120 °C. The scan parameters were 30 to 650 *m*/*z*. Interpretation of the MS spectrum was aided by Wiley and National Institute of Standards and Technology libraries of mass spectra stored in the Chemstation database (approximately 200,000 spectra) [32].

## Figures and Tables

**Table 1:** Stability of antibacterial activity of filter-sterilized potato dextrose broth culture fluids from *G. lucidum* by long term storage or boiling for one hour when compared to freshly harvested culture fluids.

Bacterial Species	Fresh Culture Fluids	90-Day-Old Culture Fluids	Boiled Culture Fluids	PDB/NA Control **
*Acidovorax avenae*	+ *	+	+	−
*Agrobacterium rhizogenes*	+	+	+	−
*Agrobacterium tumefaciens*	+	+	+	−
*Brenneria quercina*	±	±	−	−
*Burkholderia cepacia*	−	−	−	−
*Erwinia carotovora* subsp. *carotovora*	+	+	+	−
*Pseudomonas fluorescens*	−	−	−	−
*Pseudomonas syringae* pv. *syringae*	+	+	+	−
*Rathayibacter tritici*	±	±	−	−
*Xanthomonas campestris* pv. *campestris*	+	+	+	−

* Bacterial growth inhibition where: (+) = total growth inhibition; (±) = slight growth inhibition; (−) = growth of test bacterial spotted on plates containing a mixture of culture fluids and NA twofold concentrated, 1:1 ratio. ** Potato dextrose broth (PDB) and Nutrient agar 2X mixture, 1:1 ratio was used as control.

**Table 2:** Effect of protein digestion and dialysis tubing assays on antibacterial activity of culture fluids produced by *G. lucidum*.

Bacterial Species	Proteinase K Assay *		Dialysis Tubing Assay **	
	Treated CF	Non-Treated CF	Tubing CF	Beaker Diffused CF
*Acidovorax avenae*	+ ***	+	-	+
*Agrobacterium rhizogenes*	+	+	-	+
*Agrobacterium tumefaciens*	+	+	-	+
*Brenneria quercina*	NT	NT	-	±
*Acidovorax avenae*	+	+	-	+
*Erwinia carotovora* subsp. *carotovora*	+	+	-	+
*Pseudomonas syringae* pv. *syringae*	+	+	-	+
*Pantoea herbicola*	±	±	-	±
*Xanthomonas campestris* pv. *campestris*	+	+	-	+

Results are replicates of three separate experiments. NT = not tested. * Performed on nutrient agar mixed 1:1 with Proteinase K-treated PDB culture fluid (CF) or non-treated culture fluid. ** Performed on nutrient agar mixed 1:1 with tubing content of culture fluid or beaker diffused culture fluid. *** Bacterial growth inhibition where: (+) = growth inhibition; (±) = partial growth inhibition; (−) = no growth inhibition.

## Data Availability

The data presented in this study are available on request from the corresponding author.

## Sample Availability

Samples of the bioactive culture fluids of *G. lucidum* are available from the authors.

## References

[1] Bao X.-F., Wang X.-S., Dong Q., Fang J.-N., Li X.-Y. (2002). Structural features of immunologically active polysaccharides from *Ganoderma lucidum*. Phytochemistry.

[2] Su C., Sun C., Juan S., Hu C., Ke W., Sheu M. (1997). Fungal mycelia as the source of chitin and polysaccharides and their applications as skin substitutes. Biomaterials.

[3] Battle J., Ha T., Li C., Della Beffa V., Rice P., Kalbfleisch J., Browder W., Williams D. (1998). Ligand binding to the (1-->3) beta-D-glucan receptor stimulates NFkappaB activation, but not apoptosis in U937 cells. Biochem. Biophysic. Res. Commun..

[4] Diamond M., Garcia-Aguilar J., Bickford J., Corbi A., Springer T. (1993). The I domain is a major recognition site on the leukocyte integrin Mac- 1 (CD11b/CD18) for four distinct adhesion ligands. J. Cell Biol..

[5] Müeller A., Raptis J., Rice P., Kalbfleisch J., Stout R., Ensley H., Browder W., Williams D. (2000). The influence of glucan polymer structure and solution conformation on binding to (1-->3)-beta-D-glucan receptors in a human monocyte-like cell line. Glycobiology.

[6] Muller A., Rice P., Ensley H., Coogan P., Kalbfleish J., Kelley J., Love E., Portera C., Ha T., Browder I. (1996). Receptor binding and internalization of a water-soluble (1-->3)-beta-D- glucan biologic response modifier in two monocyte/macrophage cell lines. J. Immunol..

[7] Thornton B., Vetvicka V., Pitman M., Goldman R., Ross G. (1996). Analysis of the sugar specificity and molecular location of the beta- glucan-binding lectin site of complement receptor type 3 (CD11b/CD18). J. Immunol..

[8] Xia Y., Vetvicka V., Yan J., Hanikyrova M., Mayadas T., Ross G. (1999). The beta-glucan-binding lectin site of mouse CR3 (CD11b/CD18) and its function in generating a primed state of the receptor that mediates cytotoxic activation in response to iC3b-opsonized target cells. J. Immunol..

[9] Wasser S., Weis A. (1999). Therapeutic effects of medicinal properties of substances occurring in higher basidiomycete mushrooms: A current perspective. Crit. Rev. Immunol..

[10] Tanaka S., Ko K., Kino K., Tsuchiya K., Yamashita A., Murasugi A., Sakuma S., Tsunoo H. (1989). Complete amino acid sequence of an immunomodulatory protein, Ling-Zhi-8 (LZ-8). An immunomodulator from a fungus, *Ganoderma lucidum*, having similarity to immunoglobulin variable regions. J. Biol. Chem..

[11] Wang S., Hsu M., Hsu H., Tzeng C., Lee S., Shiao M., Ho C. (1997). The anti-tumor effect of *Ganoderma lucidum* is mediated by cytokines released from activated macrophages and T lymphocytes. Int. J. Cancer.

[12] Suay I., Arenal F., Asensio F., Basilio A., Cabello M., Díez M., García J., González del Val A., Gorrochategui J., Hernández P. (2000). Screening of basidiomycetes for antimicrobial activities. Antonie Leeuwenhoek.

[13] Mothana R., Jansen R., Julich W., Lindequist U. (2000). Ganomycins A and B, new antimicrobial farnesyl hydroquinones from basidiomycete *Ganoderma pfeifferi*. J. Nat. Prod..

[14] Smania A., Monache F.D., Loguericio-Leite C., Smania E.F.A., Gerber A.L. (2001). Antimicrobial activity of basidiomycetes. Int. J. Med. Mushr..

[15] Zjawiony J. (2004). Biologically active compounds from Aphyllophorales (Polypore) fungi. J. Nat. Prod..

[16] Brian D. (1951). Antibiotics produced by fungi. Bat Rev..

[17] Robles-Hernández L., Ojeda-Barrios D.L., González-Franco A.C., Hernández-Huerta J., Salas-Salazar N.A., Hernández-Rodríguez O.A. (2017). Susceptibilidad de aislados de Xanthomonas campestris pv. vesicatoria a Streptomyces y extractos bioactivos de Ganoderma. Acta Univ..

[18] Robinson J. (1995). Undergraduate Instrumental Analysis.

[19] Voet D., Voet G. (1995). Biochemistry.

[20] Mitscher L., Weinstein M., Wagman M.G. (1978). Plant-derived antibiotics. Antibiotics Isolation, Separation and Purification.

[21] Prapulla S., Subhaprada V., Karanth N., Neidleman S., Laskin A., Bennett J., Gadd G. (2000). Microbial production of oligosaccharides: A review. Advances in Applied Microbiology.

[22] Ahse U., Schroeder R. (1991). Streptomycin inhibits splicing of group I introns by competition with the guanosine substrate. Nucleic Acid. Res..

[23] Gao J.J., Min B.S., Ahn E.M., Nakamura N., Lee H.K., Hattori M. (2002). New triterpene aldehyde, lucialdehydes A-C, from *Ganoderma lucidum* and their cytotoxicity against murine and human tumor cells. Chem. Pharm. Bull..

[24] Werner G., Jolles P. (1996). Immunostimulating agents: What next? A review of their present and potential medical applications. Eur. J. Biochem..

[25] Balouiri M., Sadiki M., Ibnsouda S.K. (2016). Methods for in vitro evaluating antimicrobial activity: A review. J. Pharm. Anal..

[26] Baker C.N., Stocker S.A., Culver D.H., Thornsberry C. (1991). Comparison of the E Test to agar dilution, broth microdilution, and agar diffusion susceptibility testing techniques by using a special challenge set of bacteria. J. Clin. Microbiol..

[27] Pauli A., Schilcher H., Can Baser K.H., Buchbauer G. (2010). In vitro antimicrobial activities of essential oils monographed in the European Pharmacopoeia. Handbook of Essential Oils, Science, Technology, and Applications.

[28] Laemmli U.K. (1970). Cleavage of structural proteins during the assembly of the head of bacteriophage T4. Nature.

[29] Andrew S.M., Titus J.A., Zumstein L. (2002). Dialysis and Concentration of Protein Solutions. Curr. Protoc. Toxicol..

[30] (2002). Gel Filtration—Principles and Methods.

[31] Kuga S. (1981). Pore size distribution analysis of gel substances by size exclusion chromatography. J. Chromatogr. A.

[32] Paszczynski A., Crawford R., Funk D., Goodell B. (1999). *De novo* synthesis of 4, 5-dimethoxycatechol and 2, 5-dimethoxyhydroquinone by the brown rot fungus *Gloeophyllum trabeum*. Appl. Environ. Microbiol..

